# Obinutuzumab (GA101) is highly effective against chronic lymphocytic leukemia cells in *ex vivo* B-cell depletion irrespective of high-risk prognostic markers

**DOI:** 10.1038/bcj.2015.93

**Published:** 2015-11-13

**Authors:** L Ysebaert, E Laprévotte, C Klein, A Quillet-Mary

**Affiliations:** 1 CRCT, UMR1037 Inserm-University, Toulouse III Paul Sabatier-ERL5294 CNRS, Toulouse, France; 2 Department of Haematology, Institut Universitaire du Cancer de Toulouse, Toulouse, France; 3 Roche Innovation Center Zurich, Roche Pharma Research and Early Development, Schlieren, Switzerland

The anti-CD20 monoclonal antibody rituximab has revolutionized the management of B-cell non-Hodgkin's lymphoma and chronic lymphocytic leukemia (CLL). Addition of rituximab to the frontline standard chemotherapy (fludarabine–cyclophosphamide, FC) within the rituximab fludarabine cyclophosphamide regimen has recently shown to statistically increase both progression-free survival (PFS) and overall survival (OS) in the randomized CLL8 phase III trial of the German CLL Group.^1^ This was the first example of improved OS in first-line CLL, although restricted to those patients fit enough to receive FC chemotherapy. However, the translation of these results into the clinical setting is hampered by the fact that *ca.* one half of CLL patients are 72 years at the time of first therapy, 40% being aged more than 75 years^2^ with increased burden of comorbidities (for example, kidney function impairment) and thus not eligible for FC chemotherapy.

Based on the results of the CALGB studies^3^ and the German CLL5 trial,^4^ chlorambucil remains a treatment option of choice in those CLL patients in Europe. Despite its limited efficacy as a single agent, the favorable safety profile of chlorambucil led to its application as a backbone for reduced intensity immuno-chemotherapy in combination with rituximab and obinutuzumab (GA101). Obinutuzumab is a novel glycoengineered type II CD20 antibody that has enhanced FcgRIII affinity resulting in superior antibody-dependent cell cytotoxicity (ADCC) and antibody dependent cell phagocytosis induction as compared with rituximab; and mediates strong direct cell-death induction with a concomitant reduction in complement dependent cytotoxicity.^5^ As a type II CD20 antibody, it also shows reduced CD20 internalization in CLL samples; a mechanism that may further enhance its capability to mediate ADCC.^6^ In a series of *ex vivo-*treated CLL whole-blood samples, Patz *et al.*^7^ elucidated obinutuzumab mechanism of action against CLL cells, and demonstrated that the superior B-cell depletion of obinutuzumab observed is to a large extent due to enhanced ADCC through recruitment of (CD16)-bearing immune effector cells, as compared with rituximab at a saturating dose of 10 μg/ml. Similarily, Rafiq *et al.*^8^ confirmed the role of ADCC for B-cell depletion by obinutuzumab. The German CLL group has designed the CLL11 that randomized chlorambucil alone, rituximab–chlorambucil (R-Clb) and obinutuzumab–chlorambucil (G-Clb) in patients with CLL and coexisting conditions.^9^ Clinical data from the CLL11 trial demonstrated an acceptable safety profile with a higher rate of first infusion related reactions, but rapid and complete drop in peripheral CLL cells counts for the G-Clb arm, and improved PFS over Clb alone, as well as R-Clb (median PFS 26.7 for G-Clb vs 15.2 months for R-Clb; hazards ratio (HR), 0.39; 95% confidence interval (CI), 0.31–0.49, *P*<0.001). Most notably, the G-Clb combination induced a higher rate of complete responses and minimal residual disease negativity in a significant proportion of CLL patients, whereas minimal residual disease-negative patients in the R-Clb arm were rarely observed.^9^ These data lead to the approval of obinutuzumab in combination with chlorambucil for first-line treatment of CLL in the United States^10^ and Europe. Updated results from the CLL11 trial confirmed the previously observed OS benefit of G-Clb over Clb monotherapy (HR, 0.47; 95% CI, 0.29–0.76, *P*=0.0014).^11^ Interestingly, response to G-Clb improved outcome in all analyzed subgroups, except in patients with del(17p) where it was not significant.^9^

Here, we aimed to investigate whether rituximab and obinutuzumab B-cell depletion in *ex vivo* CLL samples is modulated by CLL-related prognostic markers, such as interphase fluorescent *in situ* hybridization (FISH) and conventional cytogenetics, immunoglobulin heavy-chain variable region mutational status (IGHV), β2-microglobulin level, recurrent somatic mutations (for example, TP53, NOTCH1 and SF3B1). All these parameters have been linked to reduce efficacy of chlorambucil monotherapy in the UK LRF CLL4 trial.^12, 13^ To date, no study has evaluated the frequency of these molecular markers across patient's age groups, but it is likely that they can decrease the efficacy of chlorambucil and other chemotherapies, making it desirable to assess the efficacy of rituximab and obinutuzumab as a single agent in a large series of *ex vivo*-treated CLL samples. For this purpose, we collected samples from 96 patients after signed, informed consent to correlate anti-CD20 B-cell depletion with modern and classical prognostic parameters (for example, FISH, immunoglobulin gene heavy variable mutational status, age, gender, Binet stage, β2-microglobulin and bulky adenopathies >5 cm) (Table 1). Fresh peripheral blood mononuclear cells were isolated from blood samples by Ficoll gradient centrifugation and subsequently cultured in high density cultures (10.10E6 cells/ml) allowing to work with viable cultures for more than 7 days.^14^ Antibody-mediated B-cell depletion was determined by enumerating trypan blue-negative, flow cytometrically CD5/CD19-positive B lymphocytes after treatment at a fixed concentration of 10 μg/ml of anti-CD20 antibodies, in RPMI+10% complement-free FCS, for 7 days.

We chose the whole-blood B-cell depletion assay as most relevant assay to compare the efficacy of obinutuzumab and rituximab as it integrates all described mechanisms of action of CD20 antibodies such as direct cell killing, ADCC, antibody dependent cell phagocytosis and complement dependent cytotoxicity. The median percentages of B-cell depletion in 96 patients were 22% with rituximab and 62.8% with obinutuzumab (*P*<0.001). Obinutuzumab had higher activity than rituximab in 83% of patients, resulting in B-cell depletion >50% in 64.6% of cases versus 16.8% for rituximab. Deletion of 11q locus (by FISH), and disruption of the p53 pathway, either by deletion of the 17p13 locus (by FISH) or *TP53* gene mutation, were parameters associated with decreased rituximab efficacy, as previously proposed elsewhere.^14^ We extended these preliminary data and demonstrate here that obinutuzumab-induced depletion is not affected in these high-risk CLL subsets (Figures 1a–c). Mutational status of *IGHV* genes and *NOTCH1* gene did not impact on either rituximab or obinutuzumab efficacy. Occurence of SF3B1 mutation was rare in our cohort, despite a trend toward a slightly decreased efficacy for rituximab, no effect for obinutuzumab was observed. This observation may be linked to the higher reported frequency of this mutation in del(11q) patients. As NOTCH1 mutations have been reported in up to one-third of trisomy 12 (tri12) patients, and tri12 being a cytogenetic abnormality with the highest CD20 expression,^15^ our results of conserved rituximab efficacy in these subgroups are not surprising. However, direct comparison between *in vitro* and *in vivo* data have to be taken with caution as NOTCH1 mutations may alter the results of rituximab fludarabine cyclophosphamide regimen in a subgroup analysis of the CLL8 trial.

Anti-CD20 MoAb activity was also reported to depend on the absolute CD20 expression level on the surface of CLL cells. Recently, CD20 internalization or CD20 shaving (by monocytic cells) after rituximab treatment, have been proposed as evasion mechanisms to antibody therapy.^6, 16^ We used the Quantibrite CD20 kit (BD QuantiBRITE fluorescent assay, BD Biosciences, Le Pont de Claix, France) to quantitatively assess CD20 expression at the surface of CLL cells from 24 patients. A cutoff value of a median CD20 expression of 8100 antibodies bound per cell was applied to categorize high versus low CD20-expressing samples (Figure 1d). CD20 expression levels was not linked to classical clinical prognostic factors, but did affect both rituximab-induced (12 vs 25%, *P*=0.37) and obinutuzumab-induced (42.5 vs 67.5%, *P*<0.05) median B-cell depletion. In both instances, obinutuzumab retained superior activity than rituximab (*P*<0.01), suggesting target modulation at the tumor surface had stronger impact on type I rather than type II antibody, as previously reported.^16^

The challenge in developing novel anti-CD20 antibodies is to demonstrate superior clinical efficacy over that achieved with rituximab. Our data suggest that based on the *in vitro* data across all prognostic subgroups of CLL patients *ex vivo*, obinutuzumab appears to work independent of (genetic) risk groups and may further qualify as a therapeutic regimen with the activity in bad prognosis CLL patients where current therapies have only limited efficacy. The data from the CLL11 study^9^ provide support for these preclinical findings supports further studies of obinutuzumab in different CLL risk groups in combination with chemotherapy- and novel-targeted reagents to ultimately improve the clinical management of CLL patients.

## Figures and Tables

**Figure 1:**
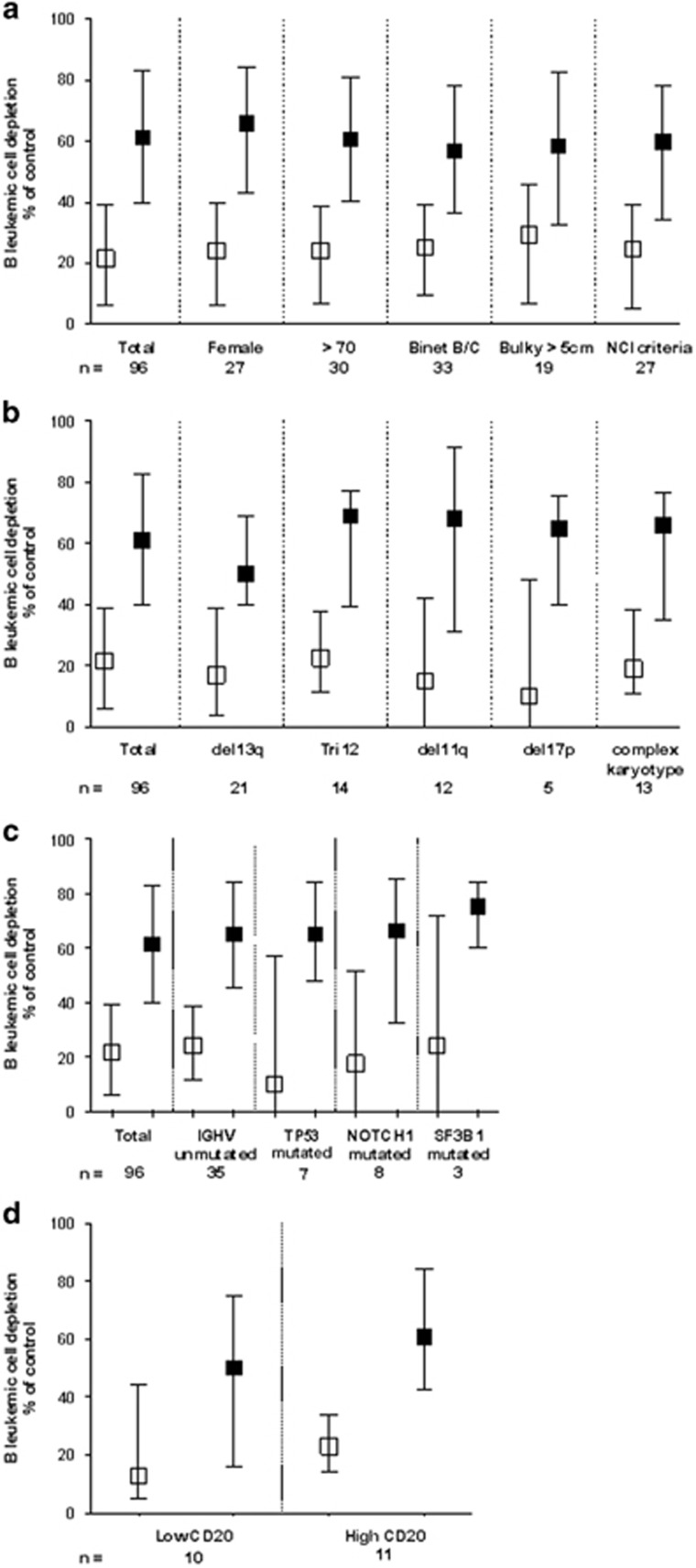
Anti-leukemic activity of a fixed saturating dose of anti-CD20 antibodies. (**a**–**c**) B-cell depletion was assessed using flow cytometry among PBMC from CLL patients, after 7 days (7d) of incubation with either rituximab (□) or GA101 (obinutuzumab) (▪). A saturating dose of 10 μg/ml was used in a complement-free medium, so results observed are due to both direct cell-death induction and antibody-dependent cell cytotoxicity. Boxes indicate means and whiskers confidence intervals. Comparisons were made using paired Student's *t*-test, all with *P*<0.05. (**d**) Depletion of leukemic cells according to CD20 surface antigen quantitative assessment. Cutoff value used (high vs low) was the median CD20 expression in a cohort of 21 patients (8100 antibodies bound per cell, using Quantibrite kits), and B-cell depletion was measured flow cytometrically after 7d in culture with 10 μg/ml of the indicated antibodies (rituximab, □ or GA101, ▪). Comparisons were made using paired Student's *t*-test, all with *P*<0.05.

**Table 1:** Clinical characteristics of CLL patients

*Characteristics*	*n*	
*Binet stage*		
A	96	65.6%
B/C	96	34.3%
Median age (years)	96	67 (41–83)
Age>70	96	30
Gender (M/F)	96	69/27
Median leukocytosis per μl	96	53 800
Bulky>5 cm	84	22.6%
		
*FISH*		
Del 13q alone/normal	85	24.7%
Del 11q	62	19.4%
Del 17p	85	5.9%
Tri12	62	22.6%
		
*Mutations*		
IGHV (MUT/UNMUT)	70	35/35
TP53	63	11.1%
NOTCH1	63	12.7%
SF3B1	62	4.7%
CD20 ABC low/high	21	10/11

Abbreviations: CD20 ABC, CD20 antibody bound per cell; del 11, 11q deletion; del 13, 13q deletion; del 17, 17p deletion; M, male; F, female; FISH, fluorescent *in situ* hybridization; tri12, trisomy 12. IGHV status: M, mutated; UM, unmutated.
